# Analysis of Different Parts of Broccoli ( *Brassica oleracea* var. *italica* ) Leaves and Retention of Functional Compounds Under Different Cooking Methods

**DOI:** 10.1002/fsn3.71157

**Published:** 2025-11-04

**Authors:** Min Gyeong Kim, Ju Hong Park, Nami Joo

**Affiliations:** ^1^ Department of Food and Nutrition Sookmyung Women's University Seoul Republic of Korea; ^2^ Department of Convergence IT Engineering Pohang University of Science and Technology (POSTECH) Pohang Republic of Korea

**Keywords:** antioxidant activities, broccoli leaves, cooking methods, phytochemicals, sulforaphane

## Abstract

The disposal of broccoli by‐products, such as broccoli leaves and stems, can potentially have harmful impacts on the environment. However, these by‐products may contain substantial amounts of phytochemical compounds with potential health benefits. In this study, the phytochemical composition and antioxidant activities of different parts of broccoli leaves, such as leaf blades and petioles, were analyzed to identify their potential use as functional food materials. Ten glucosinolates, 1 nitrile, 1 isothiocyanate, 14 hydroxycinnamic acids, 3 benzoic acids, 5 flavonoids, and 1 lignan were detected in the broccoli leaf blades, whereas 10 glucosinolates, 1 nitrile, 1 isothiocyanate, 11 hydroxycinnamic acids, 3 benzoic acids, 0 flavonoid, and 1 lignan were detected in the petioles. Antioxidant activity was consistently high in the leaf blades, indicating greater accumulation of bioactive compounds in the blades. Furthermore, broccoli leaf blades were subjected to different cooking methods (boiling, steaming, and sous‐vide) to assess changes in antioxidant activities, and sulforaphane content. Significant differences were observed depending on the cooking method, with steaming and sous‐vide methods demonstrating greater antioxidant activity than that of boiling. Although both boiling and steaming resulted in a reduction in sulforaphane content compared with that of the raw samples, the sous‐vide method led to an increase in sulforaphane content. In conclusion, broccoli leaf blades have superior phytochemical composition and antioxidant activities, and when broccoli leaf blades are cooked sous‐vide, the retention of bioactive compounds is the highest, suggesting that health functional foods can be developed through food upcycling.

## Graphical abstract

This research examines the potential of broccoli leaves, which are often overlooked as a vegetable by‐product, as a functional food ingredient. The study involved phytochemical profiling and an analysis of antioxidant activity across various leaf parts and cooking methods. The findings highlight promising nutritional benefits and suggest effective approaches for sustainable utilization.

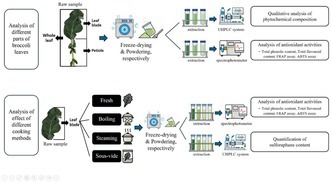

## 1 Introduction

Recently, food waste has emerged as a popular topic of discussion, especially regarding food security and climate change, due to its various negative effects and the increasing global awareness of the severity of the issue (Mokrane et al. 2023). The agri‐food industry can generate considerable amounts of waste, which can result in serious environmental problems (de los Ángeles Fernández et al. 2018). Consequently, minimizing food waste is widely acknowledged as a crucial strategy for reducing production costs and enhancing the efficiency of food systems, and it plays an important role in promoting food security and environmental sustainability (FAO 2019). These agri‐food wastes can be used as an important source of essential nutrients and functional ingredients, which can be further developed into innovative value‐added products (Domínguez‐Perles et al. 2010). According to Lau et al. (2021), agricultural by‐products, such as vegetables and fruits, can be used to improve the nutritional value of food.

Broccoli ( *Brassica oleracea* var. *italica* ), a vegetable belonging to the cruciferous family (*Brassicaceae*), is rich in health‐promoting bioactive substances, such as glucosinolates, phenolic compounds, vitamins C, B1, E, carotenoids, and selenium (Ali Redha et al. 2023; Borowski et al. 2008). However, from agricultural production to consumer distribution, cruciferous vegetables, such as broccoli, cabbage, and cauliflower, generate by‐products in the form of stems, leaves, and roots, which are considered waste during processing and discarded (Shinali et al. 2024).

Florets, which are the edible parts of broccoli, account for less than 30% of the total mass, whereas leaves and stems, which account for more than 70% of the remaining mass, generate a substantial amount of waste after the harvesting process (Zhang et al. 2017). Broccoli by‐products are treated as food waste, but broccoli leaves have a rich bioactive content compared with that of other parts of broccoli. Broccoli leaves consist of two distinct parts, the blade and petiole, each of which exhibits different anatomical and physiological functions. Broccoli leaves have higher total phenolic and flavonoid content than those of broccoli florets and seeds, demonstrating that broccoli leaves can be a potential source of glucosinolates, and have the highest content of vitamins E and K, beta‐carotene, Mn, and Ca compared with those in broccoli florets and stems (Ares et al. 2014; Le et al. 2020; Liu et al. 2018). Broccoli by‐products that are actively cultivated but not consumed have potential as functional ingredients and can be used as valuable resources for the development of nutritionally enhanced foods (Berndtsson et al. 2020).

Most vegetables are consumed after cooking, which not only contributes to their preference by softening their tissues and inactivating toxic substances but can also affect the bioavailability of bioactive substances, such as phenolic compounds (Mehmood and Zeb 2020). Glucosinolate levels in cruciferous vegetables decrease due to a variety of factors, including enzymatic action during heat treatment, heat‐induced degradation, and leaching of components into the heating medium (Oerlemans et al. 2006). However, the nutritional benefits of cruciferous vegetables, such as their anti‐cancer effects, are due to sulforaphane, a hydrolysis product of glucosinolates produced by the enzyme myrosinase (Lu et al. 2020). Myrosinase activity, which influences sulforaphane formation, is also substantially affected by heat treatment, making cooking methods a key determinant of sulforaphane formation (Yuanfeng et al. 2021).

In this study, qualitative analysis was performed using an ultra‐high‐performance liquid chromatography (UHPLC) system to determine the differences in the phytochemical profiles of different plant parts, and antioxidant activities were evaluated. Following a comparative analysis of different parts of the broccoli leaves, the parts exhibiting higher levels of bioactive compounds and antioxidant activity were selected for additional assessment, depending on the cooking method. The effects of different cooking methods on antioxidant activity and sulforaphane content were analyzed. The aim of this study was to explore the potential of different broccoli leaf parts as functional food ingredients and suggest optimal cooking methods to improve the nutritional quality of broccoli leaves.

## 2 Materials and Methods

### 2.1 Sample Preparation

**FIGURE 1:**
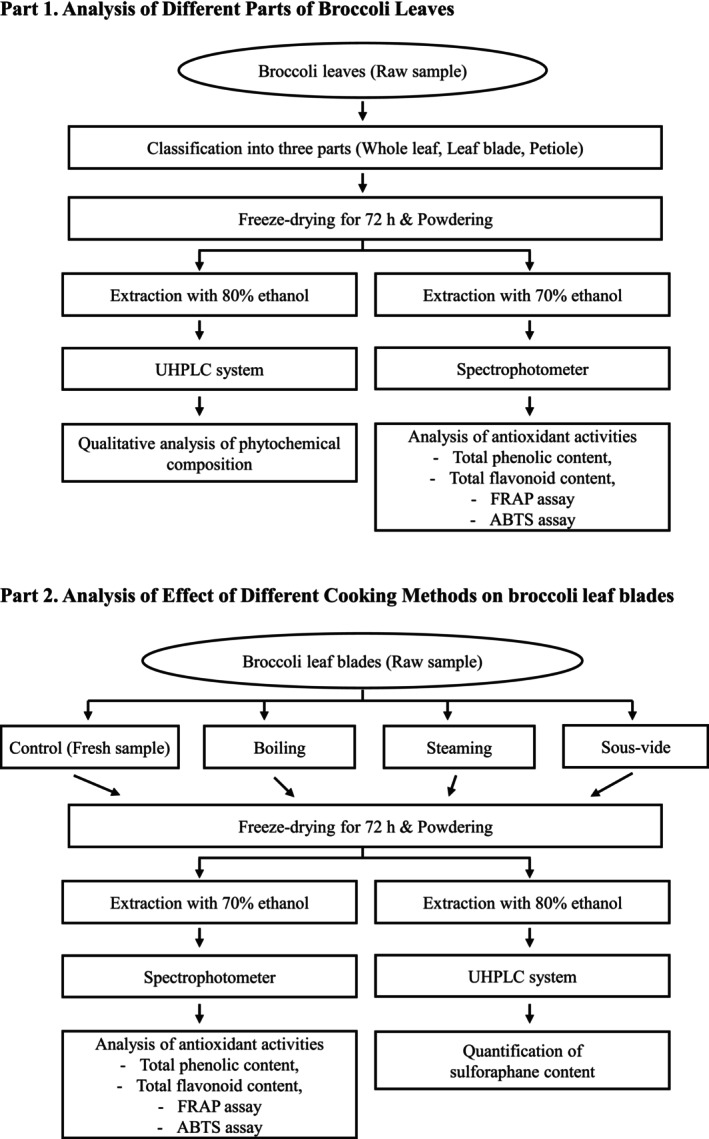
Methodological flowchart for the preparation and analysis of broccoli leaves.

The overall experimental workflow, including sample preparation and subsequent analyses, is summarized in Figure 1. Broccoli ( *B. oleracea* var. *italica* ) leaves were purchased from a local farm located in Bongseong‐ri, Aewol‐eup, Jeju‐si, Republic of Korea (33°25′06.2″ N 126°19′14.2″ E) and harvested in May 2024. They were cultivated conventionally in open fields. Initially, seeds were sown in seedling trays and grown for 30–40 days. Then, the seedlings were transplanted to the open fields and harvested after approximately 90–100 days. Broccoli leaves used in this study were the cultivar “Yuil No. 2”, an early‐maturing variety, widely cultivated in the Jeju region for its high productivity. Broccoli leaves were divided into whole broccoli leaves, blades, and petioles, and then dried at −70°C for 72 h using a freeze‐dryer (MCFD 8505, Ilshin Lab Co., Seoul, Korea). The dried samples were ground using a grinder (HB‐310, Hibell Co., Seoul, Korea) to powder through a 150‐μm mesh. The powdered samples were stored in a deep freezer (SF‐53 U, Nihon Freezer Co. Ltd., Tokyo, Japan) at −40°C and used for analysis.

### 2.2 Qualitative Analysis of Phytochemical Composition in Different Parts of Broccoli Leaves

Qualitative analysis of the phytochemical compounds in different parts of the broccoli leaves was conducted by partially modifying the method described by Lee and Joo (2022). Broccoli leaves were divided into two distinct parts: blades and petioles. The blades and petioles were freeze‐dried and powdered. The freeze‐dried powders were extracted with 80% aqueous ethanol. The extracts derived from broccoli leaves were analyzed using an Ultimate 3000 UHPLC system to identify the phytochemical compounds present in different parts of the leaves. Chromatographic separations were performed using a Waters CORTECS T3 column (2.1 mm × 150 mm × 1.6 μm, Waters, Milford, MA, USA) at a flow rate of 0.25 mL/min and a temperature of 45°C. The mobile phase consisted of solvents A (0.1% formic acid in HPLC‐grade water) and B (0.1% formic acid in acetonitrile). These phytochemical compounds were identified using a Q Exactive Plus Orbitrap mass spectrometer (Thermo Fisher Scientific Inc., Waltham, MA, USA) in both positive and negative ion modes using electrospray ionization (ESI) as the ionization method. ESI parameters were set as follows: spray voltage of 3500 V in positive mode and 3000 V in negative mode; sheath gas at 50 Arb; auxiliary gas at 10 Arb; sweep gas at 1 Arb; and ion transfer tube temperature of 320°C, with a scanning range of 100–1500 m/z. All identified phytochemical compounds were further analyzed using Scaffold Elements Viewer software (version 2.1.1, Proteome Software Inc., Portland, OR, USA).

### 2.3 Antioxidant Activities of Different Parts of Broccoli Leaves

To determine the antioxidant activities of different parts of broccoli leaves (whole broccoli leaf, blade, and petiole), and samples were freeze‐dried and powdered. After mixing the samples by different parts with 70% ethanol in a ratio of 1:10 (w/v), they were extracted at 24°C for 24 h using a shaking incubator (KSI‐200, KBT, Seongnam, Korea). After extraction, the mixture was filtered through Whatman No. 2 filter paper and the extracts were used to measure antioxidant activities.

#### 2.3.1 Determination of Total Polyphenol Content

The total polyphenol content of the samples was determined by modifying the method of Singleton et al. (1999) using the Folin–Ciocalteu method. The extracts with 0.5 mL of samples were mixed with 7.5 mL of 20% sodium carbonate (Na_2_CO_3_, DUKSAN Pure Chemicals, Ansan, Korea), followed by 2.5 mL of 2 N Folin–Ciocalteu reagent (Sigma–Aldrich Co., St. Louis, Missouri, USA). Distilled water was then added to adjust the final volume of the solution to 50 mL. The mixed solution was allowed to react in the dark for 2 h and the absorbance was measured at 765 nm using a spectrophotometer (T60UV, PG Instruments Ltd., Lutterworth, England). Gallic acid (Sigma–Aldrich Co.) was used as a standard and the total polyphenol content of the samples was calculated based on the calibration curve prepared using the same method. The total polyphenol content was expressed as μg gallic acid equivalent per gram of extract (μg GAE/g).

#### 2.3.2 Determination of Total Flavonoid Content

The total flavonoid content of the samples was measured using a modified colorimetric aluminum chloride method (Bursal and Gülçin 2011). After mixing 2 mL of the sample extract with 2 mL of 10% aluminum chloride (AlCl_3_‐H_2_O, Junsei Chemical, Tokyo, Japan), 2 mL of 1 M potassium acetate (CH_3_COOK, Junsei Chemical, Tokyo, Japan), and 11.2 mL of distilled water were added and mixed. The solution was incubated in the dark for 30 min and absorbance was measured at 415 nm. Quercetin (Sigma–Aldrich Co.) was used as the standard and the total flavonoid content of the samples was calculated based on a calibration curve created using the same method. The total flavonoid content was expressed as μg quercetin equivalent per gram of extract (μg QE/g).

#### 2.3.3 Ferric Reducing Antioxidant Power (FRAP) Assay

The FRAP of the samples was measured using the method described by Yıldırım et al. (2001). After mixing 1 mL of the extract from samples with 2.5 mL of 0.2 M sodium phosphate buffer (pH 6.6), 2.5 mL of 1% potassium ferrocyanide (III) (Sigma–Aldrich Co.) was added to the mixture. The mixed solution was placed in a water bath (WD‐06, HYSC Co., Seoul, Korea) and reacted at 50°C for 20 min. Then, 2.5 mL of 10% trichloroacetic acid (Sigma–Aldrich Co.) was added and mixed, followed by centrifugation at 3000 rpm for 10 min (Combi‐514R, Hanil Co., Daejeon, Korea). After centrifugation, 2.5 mL of the supernatant was collected, 2.5 mL of distilled water, and 0.5 mL of 0.1% (w/v) FeCl_3_ (Sigma–Aldrich Co.) were added to measure the absorbance at 700 nm. Ascorbic acid (Sigma–Aldrich Co.) was used as the standard and the FRAP values of the samples were calculated based on a calibration curve created using the same method. The FRAP value was expressed as μg ascorbic acid equivalent per gram of extract (μg AAE/g).

#### 2.3.4 ABTS Assay

The ABTS radical scavenging activity of samples was measured using the ABTS radical cation decolorization assay method (Re et al. 1999). A mixture of 1 mL of 7 mM ABTS (Sigma–Aldrich Co.) and 1 mL of 2.45 mM potassium persulfate (DUKSAN Pure Chemicals) was left in the dark for 16 h, then diluted with 70% ethanol to adjust the absorbance to 0.7 ± 0.05, which was used as the reaction solution. The extract samples (20 μL) were added to 1980 μL of the reaction solution and left in the dark for 6 min, after which the absorbance was measured at 734 nm. Trolox (Sigma–Aldrich Co.) was used as the standard and the ABTS radical scavenging activity of the samples was calculated based on the calibration curve created using the same method. The ABTS radical scavenging activity was expressed as μg trolox equivalent per gram of extract (μg TE/g).

### 2.4 Cooking Treatments of Broccoli Leaf Blades

Cooking methods for broccoli leaf blades were established based on a study by Kosewski et al. (2018), and the cooking times and temperatures were determined through several preliminary experiments. To assess the effects of different cooking methods, the fresh broccoli leaf blades were initially washed under running water, drained, and cut to a width of 5 cm and length of 3 cm. The prepared samples were then divided into four groups. One group was maintained as the fresh control, while the other three groups were subjected to different cooking treatments (boiling, steaming, and sous‐vide). For boiling, 1 L of water was boiled in a pot, and 100 g of broccoli leaf blades were added when the water started to boil (100°C), covered, and cooked for 10 min. For steaming, 1 L of water was placed in a steamer with a lid, heated to boiling (100°C), and 100 g of broccoli leaf blades were steamed for 12.5 min. For sous‐vide, 100 g of broccoli leaf blades were vacuum‐sealed in a polyethylene bag and cooked in a water bath containing 6.5 L of water at 85°C for 45 min. After cooking, the cooked samples were placed in plastic bags and cooled under running water for 3 min before drying. Subsequently, all four sample groups (fresh, boiling, steaming, sous‐vide) were freeze‐dried and powdered according to the same procedure described in Section 2.1. All powdered samples were stored in a deep freezer (SF‐53U, Nihon Freezer Co. Ltd) at −40°C for use as samples.

### 2.5 Antioxidant Activities of Broccoli Leaf Blades in Different Cooking Methods

Ethanol (70%) was used to prepare broccoli leaf blade extracts using different cooking methods for antioxidant activities. Fresh, boiling, steaming, and sous‐vide samples were freeze‐dried and powdered; 70% ethanol was added at a ratio of 1:10 (w/v), and samples were extracted using a shaking incubator (KSI‐200, KBT) at 24°C for 24 h. After extraction, the mixture was filtered through Whatman No. 2 filter paper and the extract was used as a sample to determine antioxidant activities. Total polyphenol content, total flavonoid content, FRAP assay, and ABTS assay were performed, and the antioxidant activities of broccoli leaf blades by different cooking methods were determined using the same antioxidant activity investigations as those for different parts of broccoli leaves.

### 2.6 Quantification of Sulforaphane of Broccoli Leaf Blades in Different Cooking Methods

Quantification of sulforaphane in fresh and cooked broccoli leaf blades was conducted as described by Lee and Joo (2022) with slight modifications to the method. Fresh and cooked broccoli leaf blades were freeze‐dried and powdered, and the freeze‐dried powder was extracted using 80% aqueous ethanol. Sulforaphane was quantified using a Thermo UHPLC Vanquish system with a Cortecs C18 column (2.1 mm × 50 mm, 1.6 μm, Waters Co.) at 45°C. The injection volume was 1 μL and the flow rate was 0.35 mL/min. The mobile phase consisted of 0.1% formic acid in distilled water (solvent A) and 0.1% formic acid in acetonitrile (solvent B). A gradient elution was carried out under the following conditions: 0–0.5 min, 5% B; 0.5–2.3 min, 5%–75% B; 2.3–2.5 min, 75%–100% B; 2.5–2.8 min, 100% B hold; 2.8–2.9 min, 100%–5% B; 2.9–4.0 min. Mass spectrometry (MS) analysis was performed using a Thermo TSQ Altis tandem quadrupole mass spectrometer (Thermo Fisher Scientific Inc.) equipped with a heated ESI source operating in the positive ion mode. The spray voltages were set to 3500 V in positive mode and the sheath gas pressure was set at 50 arb. The auxiliary and sweep gas pressures were set to 10 and 1 arb, respectively. The ion transfer tube temperature is 325°C and the vaporizer temperature is 350°C. Data collection and processing were conducted using Trace Finder 4.1 software.

### 2.7 Statistical Analysis

Statistical analyses of all the experimental data were conducted using SPSS Statistics (ver 23. IBM Co., Armonk, NY, USA) and the results were presented as mean and standard deviation. Additionally, a statistical significance test between samples was performed using one‐way ANOVA, and the significance between samples was evaluated at the *p* < 0.05 level. The qualitative analysis of phytochemicals was conducted using three biological replicates and other analyses were performed in three technical replicates for each treatment group. Duncan's multiple range test was performed for post hoc analysis.

## 3 Results and Discussion

### 3.1 Phytochemical Composition in Different Parts of Broccoli Leaves

#### 3.1.1 Phytochemical Composition

Qualitative analysis of the phytochemical composition in different parts of broccoli leaves was performed using a UHPLC system in both positive and negative ion modes (Figures S1 and S2). The details of the compounds identified in the qualitative analysis are listed in Table 1. The 35 identified compounds were categorized as glucosinolates, nitriles, isothiocyanates, hydroxycinnamic acids, benzoic acids, flavonoids, and lignans. Thirty‐five compounds (10 glucosinolates, 1 nitrile, 1 isothiocyanate, 14 hydroxycinnamic acids, 3 benzoic acids, 5 flavonoids, and 1 lignan) were identified in whole broccoli leaves and blades. Twenty‐seven compounds (10 glucosinolates, 1 nitrile, 1 isothiocyanate, 11 hydroxycinnamic acids, 3 benzoic acids, 0 flavonoids, and 1 lignan) were identified in petioles. The mass errors of all detected molecular ions were within 3.103 ppm, indicating that the empirical molecular formulas were consistent with the predicted precursor ions (Wang et al. 2015).

**TABLE 1:** Identification of the phytochemicals of different parts of broccoli leaves.

#	Analyte name	RT (min)	Molecular formula	Molecular weight	Adduct	Precursor ion (m/z)	Log_10_ precursor intensity			Mass error (ppm)			
							Whole leaf	Blade	Petiole	Whole leaf	Blade	Petiole	CV (%)
Glucosinolates													
1	Glucoraphanin	1.43	C_12_H_23_NO_10_S_3_	437.048	[M − H]^−^	436.041	9.431	9.650	9.676	0.316	0.105	0.067	1.40
2	Desulfo Glucoraphanin	1.46	C_12_H_23_NO_7_S_2_	357.092	[M + H]^+^	358.098	8.030	8.611	8.687	0.115	1.145	0.151	4.25
3	Glucoalyssin	2.06	C_13_H_25_NO_10_S_3_	451.064	[M − H]^−^	450.057	8.174	7.708	8.269	0.373	1.457	0.116	3.73
4	Glucoerucin	6.68	C_12_H_23_NO_9_S_3_	421.053	[M − H]^−^	420.047	7.665	8.450	8.689	0.142	0.998	0.427	6.48
5	Indolylmethyl glucosinolate	8.09	C_16_H_20_N_2_O_9_S_2_	448.061	[M − H]^−^	447.054	9.454	9.148	9.129	0.058	0.021	0.028	1.97
6	Glucosibarin	8.53	C_15_H_21_NO_10_S_2_	439.061	[M − H]^−^	438.054	7.524	6.489	7.581	0.082	0.359	1.109	8.54
7	Gluconasturtiin	10.49	C_15_H_21_NO_9_S_2_	423.066	[M − H]^−^	422.059	8.275	8.350	8.743	0.223	0.779	0.825	2.98
8	Neoglucobrassicin	12.46	C_17_H_22_N_2_O_10_S_2_	478.072	[M − H]^−^	477.064	9.373	9.392	9.494	0.352	0.230	0.057	0.69
9	Glucobrassicin	12.79	C_16_H_20_N_2_O_9_S_2_	448.061	[M + HCO_2_]^−^	493.059	9.205	7.973	8.165	0.171	0.359	0.091	7.85
10	4‐Methoxyglucobrassicin	16.39	C_17_H_22_N_2_O_10_S_2_	478.072	[M − H]^−^	477.064	9.587	9.566	9.616	0.269	0.098	0.063	0.26
Nitriles													
11	Sulforaphane nitrile	3.14	C_6_H_11_NOS	145.056	[M + H]^+^	146.064	9.290	9.884	9.289	1.845	0.472	1.109	3.61
Isothiocyanates													
12	Sulforaphane	15.27	C_6_H_11_NOS_2_	177.028	[M + H]^+^	178.035	8.398	9.208	8.343	0.411	0.719	0.008	5.60
Hydroxycinnamic acids													
13	Neochlorogenic acid	7	C_16_H_18_O_9_	354.095	[M − H]^−^	353.088	10.098	9.949	9.215	0.895	0.674	0.108	4.84
14	Caffeic acid O‐glucoside	8.03	C_15_H_18_O_9_	342.095	[M − H]^−^	341.088	8.139	7.893	7.931	0.051	0.023	0.112	1.66
15	Coumaric acid O‐glucoside	8.03	C_15_H_18_O_8_	326.100	[M − H]^−^	325.093	7.783	7.866	8.315	0.366	0.075	0.163	3.58
16	5‐Coumaroylquinic acid	9.21	C_16_H_18_O_8_	338.100	[M − H]^−^	337.093	8.960	8.726	7.947	0.033	0.522	0.433	6.21
17	Chlorogenic acid	10.69	C_16_H_18_O_9_	354.095	[M − H]^−^	353.088	8.575	8.581	8.245	0.049	0.482	0.586	2.27
18	5‐Feruloylquinic acid	11.3	C_17_H_20_O_9_	368.111	[M − H]^−^	367.103	9.500	9.243	8.253	0.489	0.054	0.252	7.31
19	Cryptochlorogenic acid	11.43	C_16_H_18_O_9_	354.095	[M − H]^−^	353.088	9.363	9.257	8.422	0.199	0.229	0.242	5.71
20	3‐Coumaroylquinic acid	12.53	C_16_H_18_O_8_	338.100	[M − H]^−^	337.093	8.381	8.212	6.422	0.102	0.500	0.318	14.15
21	1‐Caffeoylquinic acid	13.11	C_16_H_18_O_9_	354.095	[M + H]^+^	355.102	7.216	7.522	0.000	0.037	0.647	0.000	86.66
22	4‐Coumaroylquinic acid	14.07	C_16_H_18_O_8_	338.100	[M − H]^−^	337.093	8.061	7.995	0.000	0.200	0.760	0.000	86.60
23	3‐Feruloylquinic acid	15.69	C_17_H_20_O_9_	368.111	[M − H]^−^	367.104	8.815	8.669	7.713	0.018	0.294	0.710	7.13
24	Ferulic acid	17.48	C_10_H_10_O_4_	194.058	[M − H]^−^	193.050	6.727	7.653	0.000	3.103	2.753	0.000	87.14
25	4‐Feruloylquinic acid	17.74	C_17_H_20_O_9_	368.111	[M − H]^−^	367.103	7.636	7.485	6.519	0.216	0.296	1.319	8.40
26	Sinapic acid	18.41	C_11_H_12_O_5_	224.068	[M − H]^−^	223.061	7.584	8.331	6.926	2.581	1.499	1.222	9.23
Benzoic acids													
27	Benzoic acid +1O, O‐Hex	3.5	C13H16O_8_	300.085	[M − H]^−^	299.077	8.166	8.030	7.680	0.075	0.246	0.238	3.15
28	Benzoic acid +2O, O‐Hex	4.48	C_13_H_16_O_9_	316.079	[M − H]^−^	315.072	8.911	8.777	8.124	0.488	0.789	0.824	4.89
29	Benzoic acid +1O, 1MeO, O‐Hex	5.42	C_14_H_18_O_9_	330.095	[M − H]^−^	329.088	8.463	8.438	7.984	0.024	0.403	0.351	3.25
Flavonoids													
30	Kaempferol 3‐sophoroside‐7‐glucoside	11.56	C_33_H_40_O_21_	772.206	[M + HCO_2_]^−^	817.206	7.920	8.229	0.000	0.872	1.481	0.000	86.65
31	Kaempferol 3,7‐di‐O‐glucoside	14.94	C_27_H_30_O_16_	610.153	[M − H]^−^	609.146	9.068	8.351	0.000	0.260	0.658	0.000	86.82
32	Kaempferol 3,4′‐di‐O‐glucoside	17.37	C_27_H_30_O_16_	610.153	[M − H]^−^	609.147	8.076	8.578	0.000	0.342	0.877	0.000	86.72
33	Kaempferol 3‐O‐sophoroside	18.74	C_27_H_30_O_16_	610.153	[M + H]^+^	611.160	7.753	8.427	0.000	0.389	0.347	0.000	86.83
34	Astragalin	21.08	C_21_H_20_O_11_	448.101	[M − H]^−^	447.094	8.087	8.253	0.000	0.073	0.536	0.000	86.62
Lignans													
35	Acanthoside B	21.2	C_28_H_36_O_13_	580.216	[M + NH_4_]^+^	598.249	7.516	7.765	7.541	0.123	0.174	0.273	1.81

Abbreviation: CV, coefficient of variation.

Glucosinolates, known as beta‐thioglucoside‐N‐hydroxysulfates, are secondary metabolites of plants, and their chemical structures are determined by the amino acids from which they are derived, leading to their classification as aliphatic, indolic, or aromatic (Cámara‐Martos et al. 2021). The glucosinolate composition in this study was similar to that of broccoli samples reported in previous studies, although some differences were observed (Li, Zheng, et al. 2021). The glucosinolates commonly detected in both studies were 4‐methoxyglucobrassicin, glucobrassicin, glucoerucin, gluconasturtiin, glucoraphanin, and neoglucobrassicin, and glucosinolates detected in this study were desulfoglucoraphanin, glucoalyssin, glucosibarin, and indolylmethyl glucosinolate. In addition, the biological activity of glucosinolates is mainly derived from various hydrolysis products generated during plant damage (Halkier and Gershenzon 2006), and this study showed similar peak intensity of glucoraphanin, a precursor of sulforaphane, in the blades and petioles. Sulforaphane, one of the main breakdown products of glucoraphanin, is a well‐known bioactive compound derived from *Brassica* plants that has health‐promoting effects. In contrast, sulforaphane nitrile exhibits no biological activity (Radošević et al. 2017; Shokri et al. 2022). Both compounds showed higher peak intensities in the blades than in the petioles. This indicates that the broccoli leaf blade is likely the main site of sulforaphane accumulation and metabolism of sulforaphane precursors.

Hydroxycinnamic acids have potent antioxidant activities, perform various physiological functions, and have been used in the prevention and treatment of diseases associated with oxidative stress, such as atherosclerosis, cancer, and cardiovascular diseases (Teixeira et al. 2013). Except for caffeic acid O‐glucoside and coumaric acid O‐glucoside, the remaining 12 hydroxycinnamic acids showed higher peak intensities in the blades than those in the petioles; the 1‐caffeoylquinic acid, 4‐coumaroylquinic acid, and ferulic acid were not detected in the petioles. In addition, all benzoic acid compounds detected in this study showed higher peak intensities in the blades than those in the petioles. Several studies have demonstrated the role of benzoic acids as antioxidants, which are attributed to their radical scavenging ability (Harčárová et al. 2025). Thus, the higher abundance of hydroxycinnamic acids and benzoic acids detected in broccoli leaf blades suggests that the blades may exhibit higher antioxidant activity than that of petioles, indicating that broccoli leaf blades could play a more significant role in the use of functional food resources.

Four kaempferol glycosides and astragalin were detected among the flavonoids in the samples. All the identified flavonoid compounds were detected only in the broccoli leaf blades and not in the petioles, showing a high coefficient of variation of 86.60%–87.14% between samples, confirming the existence of differences among broccoli leaf parts. Similarly, Gonzales et al. (2014) showed that kaempferol glycosides were either not detected or showed significantly lower content in the cauliflower leaf petioles compared with that in the blades.

#### 3.1.2 Hierarchical Clustering Analysis of Identified Phytochemicals

**FIGURE 2:**
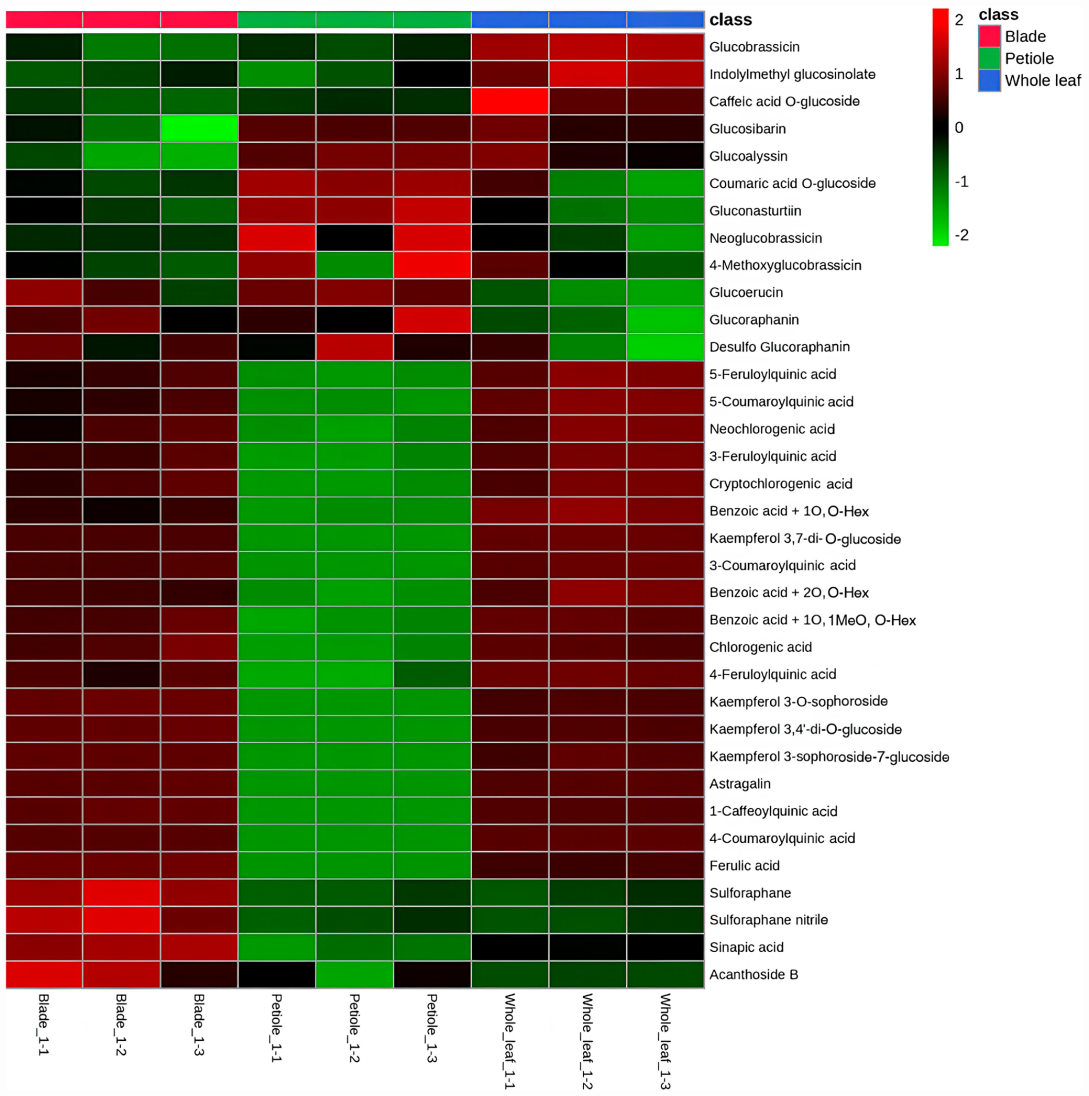
Heatmap showing identified phytochemicals in different parts of broccoli leaf.

A heatmap was created to identify differences in the phytochemical compositions of the different parts of the broccoli leaves and show the correlation between the compounds identified in the qualitative analysis (Figure 2). Most glucosinolates were strongly correlated in petioles, indicating that these compounds had relatively high peak intensities in the petioles. However, most hydroxycinnamic acids, benzoic acids, and acanthoside B showed stronger correlations in the blades than those in the petioles. Sulforaphane, another major bioactive compound in broccoli, showed stronger correlations in the blades.

Most of the compounds identified in this study were detected at higher intensities in the blades, suggesting that the differential distribution of certain compounds is likely influenced by the specific biological role of each site. Similarly, Sun et al. (2021) compared the photosynthetic contributions of the blade and petiole in cucumbers and reported that photosynthesis and carbon metabolism are primarily performed in the blade, with the petiole supporting the blade. The blade is the main metabolic site in the leaf where photosynthesis for carbon fixation occurs (Li, Kang, et al. 2021), whereas the petiole functions as a structural component that supports the leaf and optimizes its position to capture light (Filartiga et al. 2022), This may explain the lower distribution of bioactive substances in the petiole. However, the results of this study confirmed that glucosinolates, the main bioactive compounds in cruciferous vegetables, are relatively abundant in petioles. These results suggest the possibility of developing customized functional foods that maximize health benefits by considering the composition of the bioactive substances in each part.

### 3.2 Antioxidant Activities of Different Parts of Broccoli Leaves

#### 3.2.1 Total Polyphenol Content

The total polyphenol content of the whole broccoli leaf was 1171.22 μg GAE/g, while the blade had a content of 1510.39 μg GAE/g and the petiole had a content of 335.39 μg GAE/g (Table 2). The blade had the highest total polyphenol content, which was approximately 4.5 times higher than that in the petiole. Consistently, Lisiewska et al. (2006) also compared the total polyphenol content of dill parts and found that the total polyphenol content from highest to lowest was blade, whole leaf, and petiole, and the blade was four to five times higher than that of the petiole. Phenolic compounds are widely recognized for their superior antioxidant properties, and a high total phenolic content generally provides substantial health benefits (Diep et al. 2020). This indicates that the blade possessing a higher total phenolic content may demonstrate superior antioxidant activity compared with that of the petiole.

**TABLE 2:** Antioxidant activities of whole broccoli leaf, leaf blade, and petiole.

	Whole broccoli leaf	Leaf blade	Petiole	*F*
Total polyphenol content (μg GAE/g)	1171.22 ± 0.48^b^	1510.39 ± 1.27^a^	335.39 ± 0.48^c^	1579879.00***
Total flavonoids content (μg QE/g)	310.38 ± 1.49^b^	364.60 ± 3.56^a^	132.15 ± 4.08^c^	4220.36***
FRAP (μg AAE/g)	160.78 ± 0.63^b^	229.56 ± 0.06^a^	115.56 ± 0.83^c^	26991.34***
ABTS (μg TE/g)	460.17 ± 0.41^a^	460.88 ± 0.41^a^	418.50 ± 0.71^b^	6231.80***

*Note:* Different lowercase letters within a row indicate significant differences between the corresponding values (*p* < 0.05). Significance is indicated as follows: ***p < 0.001.

Abbreviations: AAE, ascorbic acid equivalent; FRAP, ferric reducing antioxidant power; GAE, gallic acid equivalent; QE, quercetin equivalent.

#### 3.2.2 Total Flavonoid Content

The total flavonoid content of different parts of the broccoli leaves is shown in Table 2. The blade had the highest total flavonoid content of 364.60 μg QE/g, followed by the whole broccoli leaf with 310.38 μg QE/g and the petiole with 132.15 μg QE/g. In the study by López‐Cervantes et al. (2013), the total flavonoid content of broccoli seeds was 33.00 μg QE/g, and the 11‐day‐old broccoli sprout, which showed the highest content during germination, was 117.26 μg QE/g, confirming that the total flavonoid content of broccoli leaves measured in this study was higher than that of broccoli seeds and sprouts. In addition, the total flavonoid content of the broccoli leaf blades was approximately three times higher than that of the petiole, which is consistent with the pattern of differences in the total flavonoid content between different parts of hydroponically grown spinach reported by Lin et al. (2014). These different antioxidant activities in different parts are likely closely related to the unique physiological functions of each plant part (Wang et al. 2024).

#### 3.2.3 Ferric Reducing Antioxidant Power (FRAP)

The FRAP assay measures the reduction of a yellow ferric tripyridyl triazine complex (Fe (III)‐TPTZ) to a blue ferrous complex (Fe (II)‐TPTZ) by an antioxidant by providing electrons (Benzie and Strain 1996). The FRAP activity of broccoli leaves was 160.78 μg AAE/g in the whole broccoli leaf, 229.56 μg AAE/g in the blade, and 115.56 μg AAE/g in the petiole (Table 2), suggesting that the blade showed the highest iron‐reducing antioxidant capacity. The differences in FRAP activity observed in this study are similar to the differences in FRAP activity reported by de Jesus Benevides et al. (2022) for Taioba, where the FRAP activity of the blade was also found to be higher than that of the petiole. A high correlation between total polyphenol content and FRAP values has been reported in a previous study (Sánchez‐González et al. 2005), which was confirmed in this study, with the highest total polyphenol content and FRAP values in broccoli leaf blades and the lowest values in the petiole.

#### 3.2.4 ABTS Radical Scavenging Activity

The ABTS radical scavenging activities of different parts of the broccoli leaves are shown in Table 2. The blade showed 460.88 μg TE/g and the whole broccoli leaf showed 460.17 μg TE/g, while the petiole showed the lowest ABTS radical scavenging activity with a value of 418.50 μg TE/g. These results confirm a previous study with Paulownia leaves (Dżugan et al. 2021), in which the Paulownia leaf blade tended to have higher ABTS radical scavenging activity than the petiole, suggesting that the higher antioxidant capacity of the blade may be a common tendency across different plants. Furthermore, in a study by García and Raghavan (2022), which assessed the ABTS radical scavenging activity of broccoli parts, the floret showed 452.17 μg TE/g and the stem showed 212.12 μg TE/g, confirming that the ABTS radical scavenging activity of the whole broccoli leaf in this study was higher than that of other parts of the broccoli.

### 3.3 Antioxidant Activities of Broccoli Leaf Blades for Different Cooking Methods

#### 3.3.1 Total Polyphenol Content

The effect of the cooking method on the total polyphenol content of broccoli leaf blades is shown in Table 3. The total polyphenol content of steaming was 1154.56 μg GAE/g, which was approximately 5% higher than that of the raw sample, and sous‐vide was 1144.28 μg GAE/g, which was approximately 4% higher than that of raw samples. Similarly, Lafarga et al. (2018) assessed the antioxidant activity of *Brassica* vegetables using cooking methods and found no significant differences in total polyphenol content between steaming and sous‐vide cooking in most samples. However, the total polyphenol content of boiling was 613.72 μg GAE/g, a decrease of approximately 44% compared with that of raw samples, resulting in a significant loss of polyphenols. The increase in total polyphenol content due to cooking, such as steaming and sous‐vide, is attributed to the release of phenolic compounds trapped in the fibers of leafy vegetables by heat, making them more available. Direct water contact cooking, such as boiling, causes polyphenols in vegetables to elute into the boiling water, leading to loss of the compounds (Adefegha and Oboh 2011; Miglio et al. 2008). In contrast, it was reported that there was no loss of total polyphenol content during sous‐vide cooking because direct contact with cooking water was prevented using vacuum packaging of the sample, thereby minimizing leaching of the components (Guillén et al. 2017).

**TABLE 3:** Antioxidant activities of broccoli leaf blades for different cooking methods.

	Fresh	Boiling	Steaming	Sous‐vide	*F*
Total polyphenol content (μg GAE/g)	1097.33 ± 0.83^c^	613.72 ± 1.27^d^	1154.56 ± 0.48^a^	1144.28 ± 0.48^b^	292843.22***
Total flavonoids content (μg QE/g)	284.64 ± 0.70^c^	188.06 ± 0.19^d^	336.71 ± 0.51^a^	323.42 ± 0.25^b^	64153.71***
FRAP (μg AAE/g)	210.86 ± 0.11^b^	109.37 ± 0.55^d^	216.52 ± 0.11^a^	204.45 ± 0.06^c^	94275.63***
ABTS (μg TE/g)	450.17 ± 1.49^c^	404.69 ± 1.09^d^	482.55 ± 1.09^a^	476.36 ± 0.71^b^	2947.59***

*Note:* Different lowercase letters within a row indicate significant differences between the corresponding values (*p* < 0.05). Significance is indicated as follows: ***p < 0.001.

Abbreviations: AAE, ascorbic acid equivalent; FRAP, ferric reducing antioxidant power; GAE, gallic acid equivalent; QE, quercetin equivalent.

#### 3.3.2 Total Flavonoid Content

Consistent with the results of total polyphenol content, steaming and the sous‐vide method increased the total flavonoid content compared with that of the raw samples, whereas boiling decreased the total flavonoid content (Table 3). Steaming increased the total flavonoid content by approximately 18%, whereas the sous‐vide method increased it by approximately 14%. Similarly, Rinaldi et al. (2021) reported that steaming and sous‐vide of pumpkin cubes increased their total flavonoid content compared with that of raw pumpkin cubes. However, in this study, boiling decreased flavonoid content by approximately 34%, confirming that boiling caused a significant loss of flavonoids. Bembem and Sadana (2013) evaluated the antioxidant activity of potato tubers according to cooking methods and found that boiling decreased the total flavonoid content, and steaming increased the total flavonoid content, which is similar to the results of this study.

#### 3.3.3 Ferric Reducing Antioxidant Power (FRAP)

The raw samples exhibited a FRAP activity of 210.80 μg AAE/g. Similarly, steaming and sous‐vide had FRAP activities of 216.52 and 204.45 μg AAE/g, respectively, representing an increase of approximately 2.7% for steaming and a decrease of approximately 3% for sous‐vide. However, the FRAP activity of boiling was 109.37 μg AAE/g, a decrease of approximately 48% compared with that of raw samples. Similar to our results, previous studies have reported a decrease in FRAP activity after sous‐vide cooking in some vegetables, such as onions and scallions; however, different trends were observed in other vegetables, such as cabbage and kohlrabi, with an increase in FRAP activity, suggesting that sous‐vide cooking may have a positive effect on FRAP activity in vegetables (Kosewski et al. 2018).

#### 3.3.4 ABTS Radical Scavenging Activity

The ABTS radical scavenging activity of raw samples was 450.17 μg TE/g, while the ABTS radical scavenging activity of steaming and sous‐vide was 482.55 and 476.36 μg TE/g, respectively, which increased by approximately 7% and 6% after cooking. However, the ABTS radical scavenging activity of boiling was 404.69 μg TE/g, a decrease of approximately 10%, and only boiling tended to decrease the ABTS radical scavenging activity among the cooking methods. Similarly, López‐Hernández et al. (2022) found that boiling broccoli florets for 10 min decreased ABTS radical scavenging activity by 47% compared with that of raw samples, while steaming for 10 min increased ABTS radical scavenging activity by 6%. However, Pellegrini et al. (2010) found that when raw broccoli florets were cooked by boiling and steaming, both methods increased the ABTS radical scavenging activity. This suggests that the ABTS radical scavenging activity of vegetables can be affected not only by the cooking method, but also by cooking conditions, such as the ratio of vegetables to water and cooking time.

### 3.4 Sulforaphane Content of Broccoli Leaf Blades in Different Cooking Methods

**FIGURE 3:**
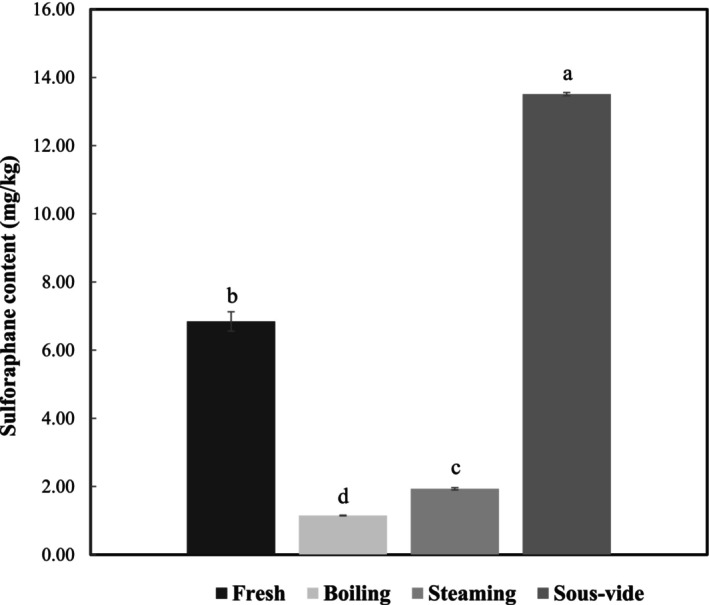
Sulforaphane content of broccoli leaf blades for different cooking methods. Results are shown as mean ± standard deviation, “a–d”: Different lowercase letters indicate significant difference between the corresponding values (*p* < 0.05).

Glucoraphanin, a glucosinolate abundant in cruciferous vegetables, such as broccoli, is hydrolyzed by an enzyme called myrosinase to form sulforaphane (Guerrero‐Beltrán et al. 2012). Several clinical studies have demonstrated the beneficial effects of sulforaphane on the human body, including anti‐cancer, antioxidant, and anti‐inflammatory effects (Vanduchova et al. 2019). However, heat treatments, such as cooking, have a significant effect on the production of sulforaphane, a hydrolysis product of glucosinolates (Pérez et al. 2014). Therefore, to determine the effect of cooking method on sulforaphane formation in broccoli leaf blades, the sulforaphane content of broccoli leaf blades cooked using different methods was assessed (Figure 3). As a result, the sulforaphane content of raw samples was 6.60 mg/kg, boiling was 1.14 mg/kg, steaming was 1.90 mg/kg, and sous‐vide was 13.51 mg/kg (Table S1). Boiling and steaming, traditional cooking methods, reduced sulforaphane content in broccoli leaf blades by 83% and 71%, respectively, whereas sous‐vide cooking increased sulforaphane content in broccoli leaf blades by 105%. Jones et al. (2010) also reported that boiling, steaming, and microwaving broccoli florets all significantly reduced sulforaphane content compared with that in raw broccoli. This decrease in sulforaphane content when cooked by boiling and steaming was most likely due to the inactivation of myrosinase by the high heat, and the greater decrease in boiling was due to the loss of glucoraphanin and sulforaphane in the boiling water (Yuanfeng et al. 2021).

The increased sulforaphane content when cooked in the sous‐vide method is due to the fact that two substances, myrosinase and epithiospecifier protein (ESP), have different thermal stability and cooking with mild heating (60°C–70°C) selectively inactivates ESP while maintaining the activity of myrosinase (Bricker et al. 2014). ESP is involved in the hydrolysis of glucosinolates, directing the hydrolysis products to epithionitrile rather than isothiocyanates, such as sulforaphane, thus preventing the formation of sulforaphane (Matusheski et al. 2004). As such, the present study suggests that sulforaphane is most abundant at temperatures between 60°C and 70°C, but other studies have shown increased sulforaphane formation when cooked at temperatures above 80°C. Blanching vacuum‐packed broccoli florets at 80°C for 10 min increased sulforaphane content (Mahn et al. 2022), and heating broccoli florets in retort pouches at 60°C, 65°C, and 80°C for 1–3 min all resulted in higher sulforaphane content than that in raw samples (Cai et al. 2020). Sous‐vide cooking of vegetables is generally recommended at temperatures above 80°C, where the cell walls are maintained, while the fiber and pectin are broken down to soften the structure, inactivate enzymes, and eliminate pathogens (Sila et al. 2009). Therefore, it is suggested that broccoli leaf blades be cooked at temperatures above 80°C when using the sous‐vide cooking method for consumption, and the results of this study confirm that sulforaphane content increases when broccoli leaf blades are sous‐vide cooked at recommended temperatures above 80°C. In conclusion, sous‐vide cooking may have increased the formation of sulforaphane because the samples were not in direct contact with water, which reduced the leaching of glucosinolates, the precursor of sulforaphane, and they were cooked in a relatively low‐temperature environment, as opposed to cooking at approximately 100°C, such as boiling or steaming, which allows the hydrolysis of glucosinolates to produce hydrolysis products in the form of isothiocyanates.

## 4 Conclusions

Phytochemicals, such as hydroxycinnamic acids, sulforaphane, and flavonoids, were detected at higher peak intensities in the blades, whereas glucosinolates were detected at higher peak intensities in the petioles. Antioxidant activities were higher in broccoli leaf blades than those in petioles. The effects of different cooking methods on broccoli leaf blades showed that steaming and sous‐vide cooking showed higher antioxidant activity than those of raw samples. Notably, the sous‐vide method increased the sulforaphane content compared with those of the raw samples, while boiling and steaming decreased the sulforaphane content. Sulforaphane content tended to increase when broccoli leaf blades were cooked at temperatures above 80°C, the recommended sous‐vide cooking temperature for vegetables. Although several studies have reported that sous‐vide cooking has a positive effect on the stability of sulforaphane, this study confirmed that this effect persists even when the cooking time is increased to approximately the optimal temperature range for sous‐vide cooking. In particular, this study confirmed that sous‐vide cooking is an optimal cooking method for stabilizing or increasing functional compounds in broccoli leaf blades. Overall, this study provides a basis for the development of functional foods using broccoli by‐products and suggests that optimal cooking methods that consider the differences in the phytochemical profiles of different parts are needed to maximize the use of broccoli leaves. However, as this study was limited to a single cultivar (“Yuil No. 2”) grown in a specific regional environment, our findings may not fully reflect the variability in phytochemical profiles caused by genetic diversity or varying environmental conditions. Therefore, further research is suggested to expand the various broccoli cultivars and include samples grown in different regions to more comprehensively assess the factors influencing the composition of bioactive compounds in different parts of broccoli leaves.

## Author Contributions

**Min Gyeong Kim:** conceptualization (lead), data curation (lead), formal analysis (equal), investigation (lead), methodology (equal), writing – original draft (lead), writing – review and editing (equal). **Ju Hong Park:** funding acquisition (equal), project administration (equal), supervision (equal). **Nami Joo:** investigation (equal), resources (equal), software (equal), validation (equal), writing – review and editing (equal).

## Ethics Statement

The authors have nothing to report.

## Conflicts of Interest

The authors declare no conflicts of interest.

## Supporting information


**Figure S1:** Total Ion Chromatograms of different parts of broccoli leaf extract in positive mode (A) Whole leaf, (B) Leaf blade, (C) Petiole.
**Figure S2:** Total Ion Chromatograms of different parts of broccoli leaf extract in negative mode (A) Whole leaf, (B) Leaf blade, (C) Petiole.
**Table S1:** Sulforaphane content of broccoli leaf blades for different cooking methods.

## Data Availability

The data that support the findings of this study are available from the corresponding author upon reasonable request.
