# Supplementary material for: Analysis of Different Parts of Broccoli ( Brassica oleracea var. italica ) Leaves and Retention of Functional Compounds Under Different Cooking Methods
Source: Food Sci Nutr. 2025 Nov 4;13(11):e71157. doi: 10.1002/fsn3.71157 (PMC12586360; doi:10.1002/fsn3.71157)
Supplement: Supplementary file 1 — Figure S1: Total Ion Chromatograms of different parts of broccoli leaf extract in positive mode (A) Whole leaf, (B) Leaf blade, (C) Petiole. Figure S2: Total Ion Chromatograms of different parts of broccoli leaf extract in negative mode (A) Whole leaf, (B) Leaf blade, (C) Petiole. Table S1: Sulforaphane content of broccoli leaf blades for different cooking methods. [file FSN3-13-e71157-s001.docx]

**Supplementary Materials:**

**Figure S1. Total Ion Chromatograms of different parts of broccoli leaf extract in positive mode (A) Whole leaf, (B) Leaf blade, (C) Petiole.**


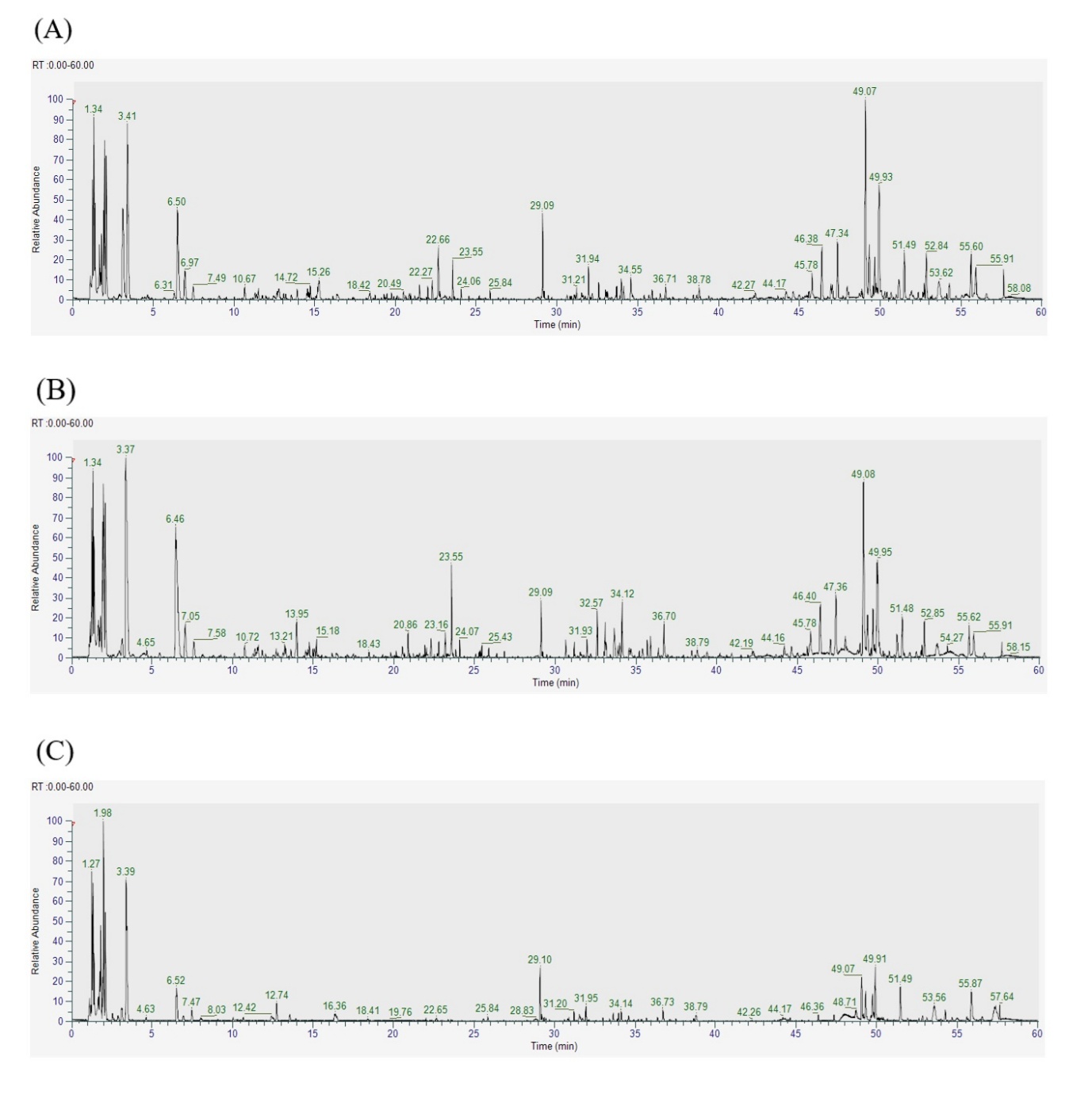


**Figure S2. Total Ion Chromatograms of different parts of broccoli leaf extract in negative mode (A) Whole leaf, (B) Leaf blade, (C) Petiole.**


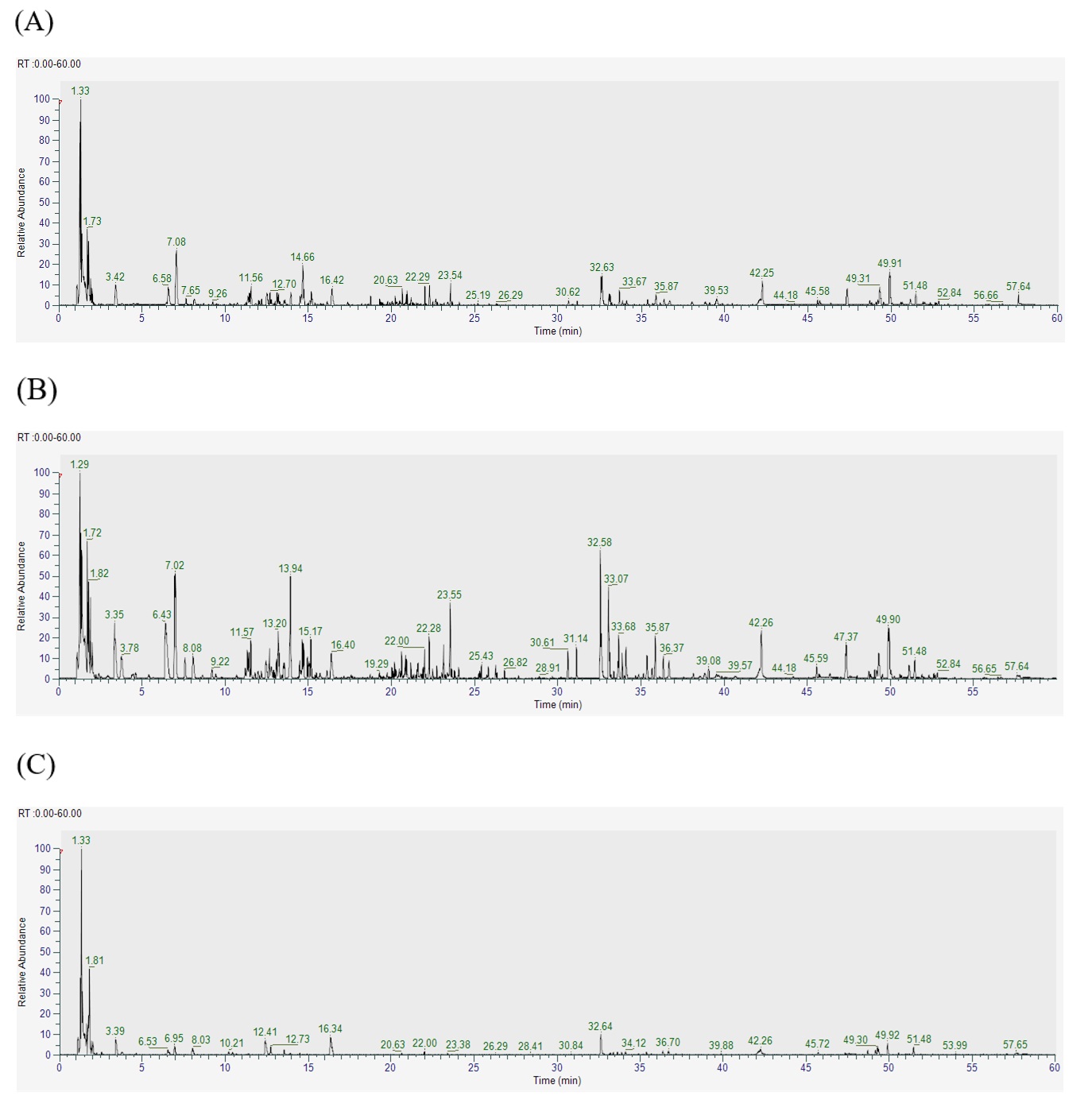


Table S1. Sulforaphane content of broccoli leaf blades for different cooking methods.

|  | Fresh | Boiling | Steaming | Sous-vide | F-value |
| --- | --- | --- | --- | --- | --- |
| Sulforaphane (mg/kg) | 6.60±0.28^b^ | 1.14±0.01^d^ | 1.90±0.03^c^ | 13.51±0.05^a^ | 4614.22^***^ |

Note: Different lowercase letters indicate significant differences between the corresponding values. (p < 0.05). Significance indicated as follows: ^***^p < 0.001.
